# Membrane Technology for the Valorization of Wood Vinegar from Grape Pomace Pyrolysis

**DOI:** 10.3390/membranes15110335

**Published:** 2025-11-05

**Authors:** Alexandre Giacobbo, Amanda de Sampaio Callegari, Mateus Torres Nazari, Valdecir Ferrari, Tania Maria Basegio, Carlos Pérez Bergmann, Marco Antônio Siqueira Rodrigues, Maria Norberta de Pinho, Andréa Moura Bernardes

**Affiliations:** 1 Post-Graduation Program in Mining, Metallurgical and Materials Engineering (PPGE3M), Federal University of Rio Grande do Sul (UFRGS), Av. Bento Gonçalves n. 9500, Porto Alegre 91509-900, RS, Brazilamb@ufrgs.br (A.M.B.); 2 Graduate Program in Technological and Environmental Chemistry (PPGQTA), School of Chemistry and Food, Federal University of Rio Grande (FURG), Av. Itália, km 8, Rio Grande 96203-900, RS, Brazil; 3 Graduate Program in Civil and Environmental Engineering (PPGEng), University of Passo Fundo (UPF), Passo Fundo 99052-900, RS, Brazil; 4 Beigrupo, Garibaldi 95720-000, RS, Brazil; 5 Department of Materials Engineering, School of Engineering, Federal University of Rio Grande do Sul (UFRGS), Av. Osvaldo Aranha 99, Porto Alegre 90035-190, RS, Brazil; 6 Department of Industrial Engineering, School of Engineering, Federal University of Rio Grande do Sul (UFRGS), Av. Osvaldo Aranha 99, Porto Alegre 90035-190, RS, Brazil; 7 Post-Graduation Program in Materials Technology and Industrial Processes, Feevale University, Rodovia RS-239, n. 2755, Vila Nova, Novo Hamburgo 93525-075, RS, Brazil; 8 Centre of Physics and Engineering of Advanced Materials (CeFEMA), Instituto Superior Técnico, University of Lisbon, Av. Rovisco Pais, n. 1, 1049-001 Lisbon, Portugal; 9 Chemical Engineering Department, Instituto Superior Técnico, University of Lisbon, Av. Rovisco Pais, n. 1, 1049-001 Lisbon, Portugal

**Keywords:** aqueous fraction from pyrolysis, wood vinegar, nanofiltration, value-added product, root growth inducer, germination index

## Abstract

The valorization of wood vinegar from biomass pyrolysis has been a significant research subject in recent years, but further studies to reduce its phytotoxicity and improve agricultural applications are still needed. This study investigates the application of ultrafiltration and nanofiltration membranes in treating the wood vinegar from grape pomace pyrolysis, aiming to valorize it. Wood vinegar treated with nanofiltration (NF270 membrane) and diluted 100 times acted as a root growth inducer in cucumber seeds, achieving a germination index of 145%. This interesting result suggests that nanofiltration is emerging as a promising technology for enhancing the value of wood vinegar, while also promoting sustainability and the circular economy in the agro-industrial sector.

## 1. Introduction

The rampant growth in global population, coupled with the rise in consumerism, has also led to a significant increase in waste generation, particularly of agro-industrial origin [1,2]. Currently, approximately 1.3 billion tons of agro-industrial wastes are generated annually worldwide [3]. This enormous amount of waste requires considerable efforts to ensure proper management, treatment, and disposal to reduce process losses and minimize associated negative impacts. Therefore, proper waste treatment and disposal are of paramount importance; however, because they are costly practices, they are often not carried out in a right manner [4].

Indeed, improper disposal of agro-industrial waste can lead to soil, water, and air contamination, posing serious risks to environmental and human health [5,6]. On the other hand, actions that promote the circular economy are appreciated because they generally reduce the costs of treating and disposing of waste and wastewater, and also promote their valorization through resource recovery, providing economic, environmental [7], and social benefits [4].

From this perspective, pyrolysis has emerged as a possible solution for agro-industrial waste [8,9]. It has proven to be a promising practice for adding value to agro-industrial biomass, with its main products being biochar, non-condensable gases, and condensable gases [10]. The latter, also known as pyrolytic liquid, is usually condensed and collected in liquid form [11] and can be separated into two distinct phases after sedimentation: a denser aqueous phase with a yellow-brownish coloration, usually called wood vinegar [12]; and a dark supernatant organic phase known as bio-oil [12,13]. It should be noted that some authors may refer to the entire liquid phase simply as crude bio-oil, liquid smoke, or other terminologies [14,15]. A variety of factors may influence the distribution, composition, and properties of non-condensable gases, biochar, and pyrolytic liquid, including the type of pyrolysis, reactor design, and process operating parameters (final process temperature, heating rate, residence time of pyrolysis volatiles, carrier gas type and flow rate), as well as the type, size, and pretreatment of the feedstock [8,11].

Most pyrolysis products already have commercial applications [16]. Bio-oil and non-condensable gases are commonly used as biofuels for energy production [15]. Biochar, in addition to being used as a biofuel, also serves as an adsorbent in water and wastewater treatment, a soil conditioner in agriculture, and an ingredient in animal feed, among other uses [16]. Conversely, wood vinegar (aqueous fraction) is regarded as a byproduct, specifically wastewater from the process, which requires proper treatment before being discharged into the environment [17]. It accounts for 15 to 35% of the pyrolytic liquid [11]. Studies show that, besides water, wood vinegar contains organic acids, furans, phenols [17] and other organic compounds [8,18]. Given the challenges related to its treatment, and its rich composition in organic substances derived from bio-oil, wood vinegar has been a focus of research for its valorization. It has been explored as an insect repellent [19,20], an animal feed additive [21,22], an antimicrobial agent [12,23], a pesticide [24], a pest and disease control agent [10,25], a soil conditioner [26,27], a root growth enhancer [28], a plant development and growth stimulant [29], and a method to increase fruit plant productivity [30,31].

Indeed, various studies have explored a range of potential applications for wood vinegar, but none of these findings are yet conclusive, and further research is necessary. It is important to note that some studies have yielded conflicting results. For example, while Griebeler et al. [28] reported that wood vinegar from the pyrolysis of *Acacia mearnsii* De Wild, at concentrations between 3.5% and 4.5%, acted as a rooting enhancer for subtropical eucalyptus clones, Anggrayni et al. [32] found that wood vinegar, derived from the pyrolysis of coconut shell and acacia wood residues, was phytotoxic to banana trees at concentrations above 2%. Both studies primarily attributed these effects to the phenolic compounds present in the wood vinegar analyzed. In this context, as Chai et al. [33] mentioned, some pretreatment may be necessary to remove undesirable substances or to enrich beneficial compounds, thereby improving certain applications and increasing the value of wood vinegar.

Recent studies have demonstrated the ability of membrane technologies, such as ultrafiltration (UF) and nanofiltration (NF), in reducing the total phenolic content in agro-industrial extracts and wastewater [34,35,36,37]. These technologies have also been shown to be effective in fractionating macromolecules and small organic molecules [38,39], highlighting their potential as promising options for decreasing the phytotoxicity of wood vinegar and adding value to this byproduct.

While UF membranes have applications in the separation and fractionation of macromolecules, as they have pore sizes ranging from 1 to 100 nm [40] and molecular weight cut-offs (MWCOs) from 1000 to 350,000 Da [41], NF membranes are mainly used for the fractionation of small organic solutes and salts, as they have MWCOs between 200 and 1000 Da. In addition, NF membranes usually exhibit negative charge at neutral pH and exert high rejection coefficients for divalent salts (>90%) and moderate rejection coefficients for monovalent salts (40 to 90%) [7]. The separation capacity and productivity of membranes are primarily determined by their pore size, which is closely related to their MWCO. Nevertheless, other factors such as membrane material, solute–membrane and solute–solute interactions (regarding surface charge, hydrophobicity, and molecular shape), and operating conditions (e.g., pressure, temperature, feed flow rate, volumetric concentration factor) also play a crucial role [7,41,42].

In fact, membrane technologies have gained prominence over traditional separation methods due to their inherent advantages, including low energy consumption, mild operating conditions, absence of additives, high separation efficiency, easy scalability, and improved safety [43,44]. Taking into account these aspects, this study investigated the use of UF and NF to add value to wood vinegar produced from grape pomace pyrolysis—a widely generated agro-industrial waste in wine regions—by reducing its phytotoxicity and increasing the germination index in cucumber seeds. To minimize membrane fouling, we evaluated three commercial hydrophilic membranes commonly used for the removal of phenolic compounds, the target compounds of this study: two polyethersulfone (PES) UF membranes with MWCOs of 3000 and 10,000 Da, and a thin-film composite NF membrane with a semi-aromatic polyamide (PA) active layer and an MWCO of 400 Da.

## 2. Materials and Methods

### 2.1. Wood Vinegar

Grape pomace from a winery in Serra Gaúcha, RS, Brazil, was dried at 120 °C, ground, sieved through 5 and 10 mm mesh screens, and then pyrolyzed. Pyrolysis was carried out in a laboratory-made Auger pyrolyzer, with nitrogen injection at a flow rate of 2 L min^−1^, a biomass feed rate of approximately 3 kg h^−1^, and a temperature of 350 °C. The smoke was condensed and collected as a pyrolytic liquid, which was sent to a separatory funnel, yielding an oily fraction and the aqueous fraction (wood vinegar) that is the subject of this study.

### 2.2. Membrane Filtration Experiments

Three commercial membranes covering a wide range of molecular weight cut-offs (MWCO) were evaluated, comprising one nanofiltration membrane and two ultrafiltration membranes. The main characteristics of the membranes are presented in Table 1.

Permeation runs were performed in laboratory flat-cell units with a 14.5 cm^2^ membrane surface area, as thoroughly described in previous studies [36,55]. The membranes were initially washed by circulating a sodium hydroxide solution at pH 10 at 30 °C and 1 bar pressure for 30 min. They were compacted by circulating deionized water pressurized to 5 bar for two hours to avoid pressure effects on the membrane structure in subsequent experiments. The membranes were then characterized for permeability to pure water at pressures of 2 to 5 bar, as described by [56]. The experiments were conducted in full recirculation mode—in which permeate and retentate continuously return to the feed tank—with 5 L of wood vinegar at a temperature of 25 °C, a feed flow rate of 200 L h^−1^, and a pressure of 4 bar. The experiments lasted approximately six hours, and during this time approximately 100 mL of permeate from each membrane was collected for analysis. The permeation flux (*J*) was monitored throughout the experiments. It was calculated using Equation (1), where *m* is the permeate mass (kg), *A* is the membrane surface area (m^2^), and *t* is the permeate collection time (h). Between each experiment, the membranes were washed by circulating a pH 10.5 sodium hydroxide solution at a flow rate of 200 L h^−1^ and a temperature of 30 °C, until the permeability to pure water of each membrane reached at least 90% of the initial value. Each experiment was conducted at least twice, and the results are presented as mean ± standard deviation.

(1) $$J=\frac{m}{A\;t}$$

### 2.3. Phytotoxicity Assays

Phytotoxicity assays were performed using seeds of cucumber (*Cucumis sativus*), following the methodology described by [57,58].

In these assays, 10 cucumber seeds were placed on filter paper inside a 90 mm diameter Petri dish and exposed to 5 mL of each sample: deionized water (control group), and samples of the feed and permeates from the different membranes assessed. The plates were sealed with Parafilm and incubated in a germination chamber for 120 h at 25 °C. After the incubation period, the germination index (*GI*) was determined using Equation (2) [59]. It was calculated based on the results of relative seed germination (*RSG*), where seeds with root lengths greater than 1 mm were considered germinated, and relative root growth (*RRG*), whose root length was measured using a digital caliper. The sample is considered phytotoxic when the *GI* is less than 80% compared to the control [60]. These assays were performed in triplicate, using both pure samples and samples diluted 1:100.

(2) $$GI\;\left(\%\right)=\frac{RSG\times RRG}{RSGc\times RRGc},$$

where *RSG* is the number of germinated seeds on the sample plate, and *RRG* is the root growth on the sample plate. *RSGc* and *RRGc* represent the average number of germinated seeds and the average root growth in the control group, respectively.

### 2.4. Analytical Methods

Wood vinegar was characterized for its total phenolic compound (TPC) content by a colorimetric method using a UV-Vis spectrophotometer (PG Instruments, Lutterworth, UK), measuring absorbance at 280 nm [61] and expressing the results as mg L^−1^ of gallic acid equivalent (GAE). A TOC-L_CPH_ carbon analyzer (Shimadzu Scientific Instruments Inc., Kyoto, Japan) was employed to measure total organic carbon (TOC).

## 3. Results and Discussion

**Figure 1:**
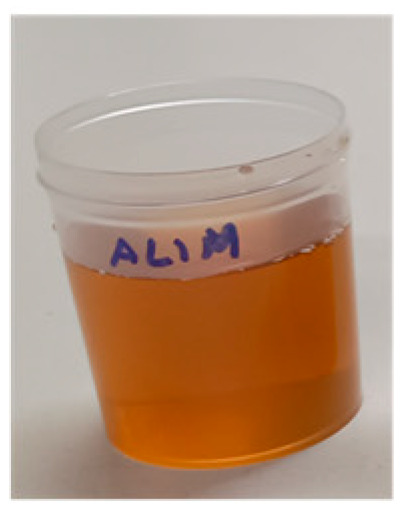
Wood vinegar obtained in this study.

Pyrolysis of grape pomace produced non-condensable gases, biochar, and crude bio-oil that was subjected to sedimentation to separate the wood vinegar, which is the subject of this study. The resulting wood vinegar has a brownish color (Figure 1), a slightly acidic pH (6.4), and 4630 mg L^−1^ GAE of total phenolic compounds; these characteristics are similar to those reported in other studies [62,63,64].

**Figure 2:**
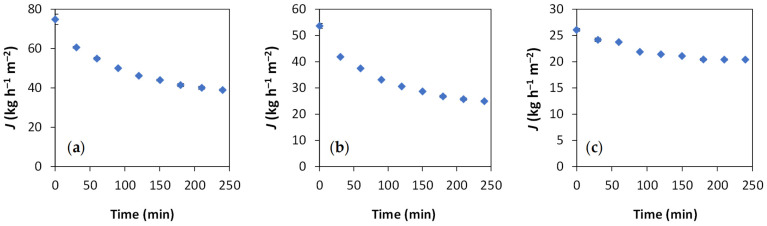
Permeate fluxes as a function of operating time in wood vinegar filtration experiments with membranes VT (**a**), ST (**b**) and NF270 (**c**).

Figure 2 illustrates the permeate flux behavior over time during the membrane filtration experiments conducted with wood vinegar. It shows that the permeate flux begins at higher levels and gradually decreases, stabilizing after about 200 min. At the end of the tests, the VT, ST, and NF270 membranes exhibited fluxes of 39, 25, and 20 kg h^−1^ m^−2^, respectively. This decline in flux is typical of phenomena like concentration polarization and membrane fouling [34,38]. Although the VT and ST membranes (Figure 2a,b) showed the highest initial permeate fluxes, they also experienced the greatest declines, with reductions of about 50%, indicating more severe fouling. Conversely, the NF270 membrane (Figure 2c) showed the smallest flux reduction over the experiments (ca. 22%) and, therefore, the least fouling. Other studies [42,65] have reported polar interactions between phenolic compounds and PES membranes, leading to the adsorption of these substances onto the membranes and, consequently, fouling, which results in a loss of productivity. In contrast, the NF270 membrane has a semi-aromatic polyamide active layer, a more negative zeta potential, and greater hydrophilicity (lower contact angle) (see Table 1); these features significantly contributed to a lower incidence of fouling [49]. Furthermore, other studies have also indicated that the polyamide membranes are less susceptible to fouling by phenolic compounds compared to PES membranes [41,42,65,66]. The MWCO of the membranes may also have influenced the loss of productivity. Minhalma et al. [67] stated that membranes with larger MWCO are more prone to fouling and reductions in the permeate flux, which aligns with our observations. These results are indeed encouraging, as they are consistent with flux levels reported in the literature [41].

**Figure 3:**
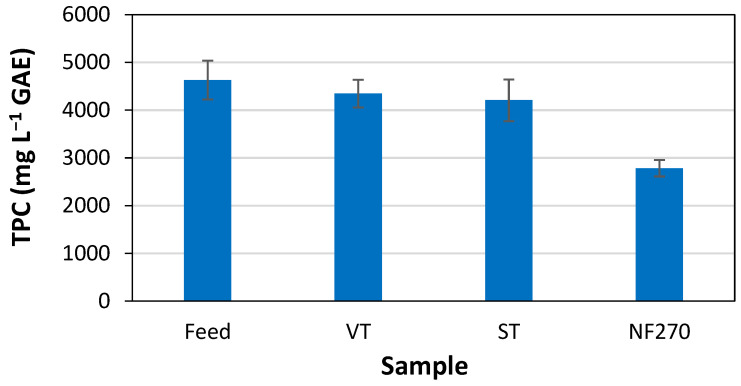
Total phenolic compound (TPC) content in raw wood vinegar (feed) and in permeates from VT, ST, and NF270 membranes.

**Figure 4:**
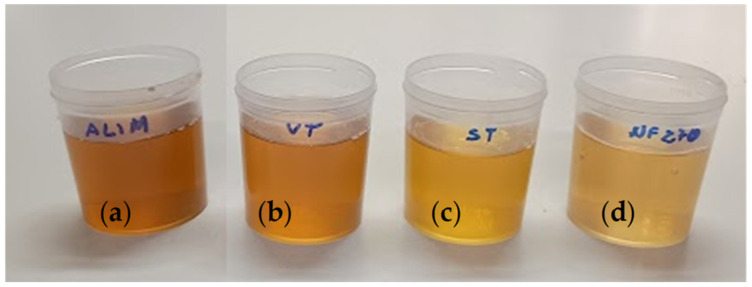
Images of wood vinegar samples. Raw wood vinegar used as feed in membrane filtration experiments (**a**), and permeates from the VT (**b**), ST (**c**), and NF270 (**d**) membranes.

The content of phenolic compounds in the feed samples and in the permeates from the different membranes evaluated are displayed in Figure 3. As previously mentioned, the raw wood vinegar, used as the feed in the membrane filtration experiments, had a TPC of 4630 mg L^−1^ GAE. The permeates obtained from the VT, ST, and NF270 membranes yielded TPC of 4350, 4206, and 2784 mg L^−1^ GAE, respectively. These results indicate that the membranes were capable of removing approximately 6.0%, 9.1%, and 40% of the phenolic compounds from the wood vinegar used as the feed solution. Considering the MWCO of the membranes evaluated, we can infer that the vast majority of phenolic compounds in the wood vinegar have a molecular weight of less than 3 kDa. This inference is based on the fact that the VT membrane, which has an MWCO of 3 kDa, rejected only 6.0% of the phenolic compounds. Furthermore, based on the TPC rejection results for the NF270 membrane, approximately 60% of the phenolic compounds in the wood vinegar likely have a molecular weight lower than 400 Da. Furthermore, the color of the permeate samples intensifies with increasing membrane MWCO, as illustrated in Figure 4. Notably, the color of the permeate sample from the NF270 membrane (which has the lowest MWCO and the highest TPC rejection) is much less intense than that of the wood vinegar used as the feed solution and also less intense than that of the permeate samples from the other two membranes. This suggests that a portion of the phenolic compounds may be responsible for imparting color to the wood vinegar, as stated in previous literature [68].

**Figure 5:**
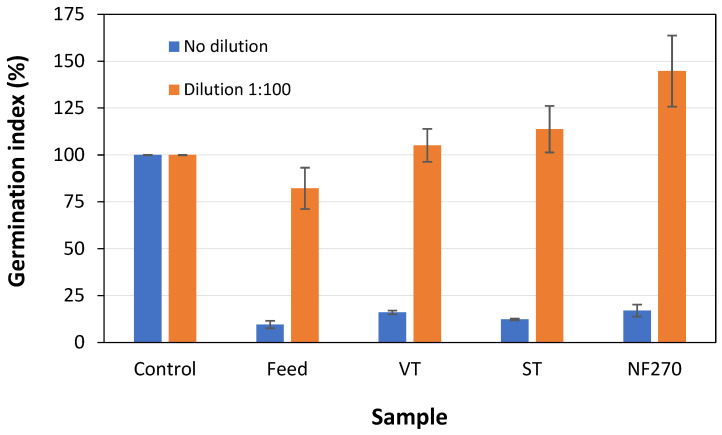
Germination index of cucumber seeds. Tests performed with undiluted and 100-fold diluted samples. Deionized water was used as a control group.

The results of the germination index in cucumber seeds are presented in Figure 5. It can be seen that the wood vinegar and the membrane permeates evaluated were phytotoxic to the cucumber seeds, since these samples exhibited a much lower *GI* than those achieved with the control group (deionized water). However, membrane technologies reduced the phytotoxicity of the wood vinegar, since there was an increase in the *GI* of the seeds treated with the membrane permeates compared to those treated with raw wood vinegar (feed solution). While the *GI* obtained in the treatment with the feed solution was only 9%, that of the treatment with the NF270 membrane permeate almost doubled, reaching 17%. These results are likely associated with the phenolic compound content of the samples. In tests conducted with banana plants as the test organism, Anggrayni et al. [32] attributed the phytotoxicity they found to the presence of phenolic compounds in the wood vinegar. Likewise, Iacomino et al. [69] reported a decrease in the total yield of greenhouse-grown strawberry plants when treating them with wood vinegar, which was considered phytotoxic to the cultivar at concentrations ≥ 5%.

Nevertheless, Figure 5 also shows that treatments performed with samples diluted 100 times yielded completely different results from those observed with the undiluted samples. At a 100-fold dilution, all samples were non-phytotoxic to cucumber seeds (*GI* > 80%) [43]. The feed solution diluted exhibited an average *GI* of 82%, and the membrane permeates had higher *GI*s than the control group. This higher *GI* was particularly observed in treatments with the NF270 membrane permeate, which reached *GI* values as high as 145%. In this case, the NF270 membrane permeate acted as a root growth inducer. This may have occurred because the NF270 membrane, in addition to removing a greater amount of phenolic compounds from the wood vinegar, also removed those of higher molecular weight, suggesting that higher molecular weight phenolic compounds may be more phytotoxic than lower molecular weight ones.

According to Zhang et al. [70], wood vinegar can act as a natural elicitor. However, previous studies [46] suggest that wood vinegar may have antagonistic effects, depending on the concentration used and the plant species evaluated. Accordingly, wood vinegar also exhibits herbicidal potential [71,72]. On the other hand, when diluted, wood vinegar stimulates seed germination, root and seedling growth [69,73,74], representing a promising use in the agricultural context. Therefore, it is important to assess its effects on plants to guide its application, either as a weed control agent or as a plant growth promoter.

By converting grape pomace through pyrolysis into a valuable agricultural input, a direct contribution to environmental sustainability, food security, and responsible resource management is achieved. In this context, it aligns with several United Nations Sustainable Development Goals (SDGs) [75], particularly SDG 2 (Zero Hunger; through higher yields and sustainable farming), SDG 3 (Good Health and Well-being; by reducing agrochemical use and minimizing exposure to harmful compounds), SDG 12 (Responsible Consumption and Production; by valorizing biomass by-products within a circular economy framework), and SDG 15 (Life on Land; by enhancing soil health and stimulating beneficial microbial communities). Therefore, wood vinegar valorization through membrane technology represents an intersection of innovation and sustainability, transforming waste into a value-added agricultural product while supporting ecological balance and global food security.

## 4. Conclusions

Wood vinegar obtained by grape pomace pyrolysis was phytotoxic to cucumber seeds. Nevertheless, the evaluated membrane technologies, especially nanofiltration, showed promise in reducing this toxicity. The permeate obtained with the NF270 nanofiltration membrane, when diluted 100-fold, demonstrated a cucumber seed germination index of 145%, thus acting as a root growth inducer. These results indicate that nanofiltration emerges as a promising alternative for valorizing liquid waste byproducts from biomass pyrolysis. In summary, this membrane technology offers significant potential for transforming liquid byproducts from biomass pyrolysis into valuable resources, thereby enhancing the sustainability and economic viability of biomass thermochemical conversion processes.

Indeed, this study serves as a starting point for the application of membrane technology in the valorization of wood vinegar, which, incidentally, is a byproduct that remains under-researched. Therefore, further studies are needed to leverage this application, and in-depth analysis of the following aspects is recommended: (i) investigation of membranes with intermediate MWCO when compared to those studied here, aiming to fractionate molecules of interest; (ii) investigation of membranes made of other materials and testing under other operating conditions, aiming to minimize membrane fouling; (iii) more detailed characterization of the entire aqueous fraction, including not only the identification of phenolic compounds, but also the main compounds of interest and the toxic substances; and (iv) phytotoxicity testing on different seed species and testing at different stages of plant growth, to allow for a broader comparison in terms of biological diversity and plant response.

## Figures and Tables

**Table 1:** Characteristics of the selected membranes.

Parameter	Membrane Type		
	NF270	VT	ST
Membrane active layer	Semi-aromatic Polyamide ^a,b,e^	Polyethersulfone ^f^	Polyethersulfone ^h^
MWCO (Da)	400 ^c^	3000 ^f^	10,000 ^h^
pH operating range	3–10 ^a^	3–9 ^f^	3–9 ^h^
Maximum operating pressure (bar)	41 ^a^	8.3 ^f^	8.3 ^h^
Zeta potential (mV)	−22 ^d^; −29 ^b^	n.a.	−9.6 ^i^
Contact angle (°)	30 ^e^	n.a.	59 ^i^
Pore radius (nm)	0.44 ^b^	1.52 ^g^	7.84 ^j^
Manufacturer	FilmTec—DuPont	Synder Filtration	Synder Filtration

n.a.: not available; ^a^ [45]; ^b^ [46]; ^c^ [47]; ^d^ [48]; ^e^ [49]; ^f^ [50]; ^g^ [51]; ^h^ [52]; ^i^ [53]; ^j^ [54].

## Data Availability

The data that support the findings of this study are available from the corresponding authors upon reasonable request.
